# Atypical Mucin Expression Predicts Worse Overall Survival in Resectable Pancreatic Ductal Adenocarcinoma

**DOI:** 10.1155/2022/7353572

**Published:** 2022-07-21

**Authors:** Yunzhen Qian, Yitao Gong, Yu Liu, Yusheng Chen, Ruijie Wang, Zhengjie Dai, Xuan Zou, Yesiboli Tasiheng, Guopei Luo, Xuan Lin, Xu Wang, He Cheng, Xianjun Yu, Chen Liu

**Affiliations:** ^1^Department of Pancreatic Surgery, Fudan University Shanghai Cancer Center, Shanghai 200032, China; ^2^Department of Oncology, Shanghai Medical College, Fudan University, Shanghai 200032, China; ^3^Shanghai Pancreatic Cancer Institute, Shanghai 200032, China; ^4^Pancreatic Cancer Institute, Fudan University, Shanghai 200032, China

## Abstract

**Background:**

Pancreatic ductal adenocarcinoma (PDAC) displays a typical mucin expression pattern which is characterized by MUC1 positive, MUC2 negative, and MUC5AC positive. More and more evidences show that mucins are involved in the development of pancreatic diseases. However, the relationship between mucin expression and prognosis of PDAC patients has been controversial in the past decades; therefore, we aim to figure out the association of mucin expression with survival in PDAC patients who underwent radical resection.

**Methods:**

We performed immunohistochemistry (IHC) to detect the expression of MUC1, MUC2, and MUC5AC in resected PDAC specimens from Shanghai Cancer Center, Fudan University (FUSCC, *n* = 427) and obtained the corresponding clinical statistical data for retrospective study. Kaplan-Meier methods and Mantel-Cox tests were used to compare the survival curves, and the Cox regression model was employed for multivariate analyses to determine the independent risk factors. Survival analysis was also performed in the Queensland Centre for Medical Genomics (QCMG, *n* = 70) PDAC cohort to verify the conclusion.

**Results:**

Both the FUSCC cohort and the QCMG cohort demonstrated that MUC1 absence was significantly correlated with worse overall survival (OS). The presence of MUC2 showed marginal significance in predicting shorter OS of PDAC patients, while MUC5AC had no prognostic value. In the FUSCC cohort, MUC1 absence was associated with increased proportion of stage III PDAC (*p* = 0.011), and MUC1 absence and MUC2 presence were associated with tumour perineural aggression (*p* = 0.011 and *p* = 0.030, respectively). Multivariable adjusted hazard ratios (HRs) for mortality of MUC1 and MUC2 were 0.492 (95% CI: 0.274-0.883, *p* = 0.017) and 1.596 (95% CI: 1.061-2.401, *p* = 0.025), respectively.

**Conclusions:**

MUC1 absence or MUC2 presence is independently associated with poor OS among patients with resectable PDAC. MUC5AC absence tended to be associated with short-term death.

## 1. Introduction

Pancreatic ductal adenocarcinoma (PDAC) is a notorious malignancy with a poor survival rate. In the past few decades, the incidence rate of PDAC has been rising and is expected to become the second top cancer killer in 2030 [1]. However, the prognosis of PDAC was barely improved; therefore, detecting prognostic factors and potential therapy targets is of great importance for PDAC. Notably, PDAC is characterized by aberrant glycosylation and stroma formation.

Mucins are a group of O-glycoproteins, which are composed of a protein backbone and a diversity of carbohydrate side chains with abundant threonine and serine [2], and could be classified as secretory mucins and membrane-bound mucins [3]. Alteration of mucins is commonly present in epithelial neoplastic lesions such as PDAC, breast cancer, ovarian cancer, and colon cancer [4]. Mucins are important markers for identifying tumour lineage and differentiating tumour subsets [5]; some mucin expression patterns indicate specific precursor lesions [6] for PDAC, such as mucinous cystic neoplasms (MCN) and intraductal papillary mucinous neoplasms (IPMN). Remarkably, mucins are not only bystanders but also culprits in the generation and development of PDAC; in addition to its prognostic value, there is increasing evidence indicating that mucins are involved in inflammation and oncogenesis.

MUC1, also known as carbohydrate antigen 15-3 (CA15-3), is a membrane-bound mucin predominantly expressed in normal pancreatic duct cells, and PDAC usually has significantly elevated levels of sialylated MUC1 [7]. The secretion of MUC2 forms an insoluble mucous gel barrier, which is usually identified as a tumour suppressor [8]. The expression of MUC2 in both normal pancreatic tissue and PDAC is rare. In contrast, another gel-forming mucin MUC5AC is abundantly overexpressed in PDAC tissues and potentiates the oncogenic signalling pathway [9]. Accordingly, these aberrantly glycosylated mucins can be recognized as tumour-associated antigens and can be used as useful predictors of adjuvant therapeutic efficacy [10, 11] and potential targets for cancer therapy [12]. For example, MUC1 has epitopes for cytotoxic T lymphocytes [13] and is expected to develop cancer vaccines or chimeric antigen receptor T cells [14].

There exists a subgroup with atypical mucin expression pattern, accounting for approximately 20% to 30% of PDAC patients. Previous studies reported inconsistent conclusions about the association between mucin expression and PDAC patients' survival. Investigating the subgroup of PDAC patients is crucial to develop precision oncology and targeted therapy in PDAC; hence, it is important to determine the correlation between atypical mucin expression and PDAC development.

In this study, we used IHC to determine the expression of MUC1, MUC2, and MUC5AC in resected PDAC tissues and analyzed its correlation with clinicopathologic characteristics and postoperative survival of patients in the FUSCC PDAC cohort. Our conclusions were validated by analyzing the QCMG PDAC cohort. The major purpose of this study was to find out the correlation between mucin expression and OS of PDAC patients in order to identify risk factors for PDAC management and potential targets for future treatment. The additional aim is to investigate the association between mucin expression and clinicopathological features.

## 2. Methods

### 2.1. Online Dataset and Specimens

Open access data (RNA expression level and survival data) was sourced from the QCMG dataset [15] using cBioPortal (http://www.cbioportal.org/). Only 70 samples with definite pathological diagnosis of PDAC were included.

Tumour specimens were obtained from 427 Chinese patients who underwent radical surgical resection and had definite postoperative pathological diagnosis of PDAC from March 2012 to May 2017 in the Department of Pancreatic Surgery Shanghai Cancer Center, Fudan University, China. Patients with the following features were excluded: (1) patients without follow-up data, (2) patients with multiple primary malignancies or secondary malignancies, (3) patients with pancreatic neoplasms other than PDAC, (4) patients with haematological disorders, and (5) patients died within 90 days due to surgical complications.

### 2.2. Baseline and Clinicopathological Characteristic Data

Information about age, gender, tumour grade, tumour-node-metastasis (TNM) stage, tumour location, status of perineural infiltration, vascular invasion, diabetes mellitus history, carbohydrate antigen 19-9 (CA19-9) level, and adjuvant therapy history were acquired from the patients' medical history from FUSCC. CA19-9 levels were collected according to the preoperative serum tests. Tumour grade was assessed according to the fifth edition of the WHO Classification of Tumours [16] and was reviewed by expert pathologists. Tumour-node-metastasis stage was determined based on the American Joint Committee on Cancer (AJCC), 8th edition [17], and tumour size, numbers of metastatic lymph nodes, and status of metastasis were recorded according to the histological pathological reports of resected specimens. OS was defined as the length of time (days) from diagnosis to death from any cause (or the last reliable follow-up). Follow-up ended in March 2021.

### 2.3. Immunohistochemistry

Tumour specimens acquired from operation were fixed in 10% formalin and embedded with paraffin. Then, tissue blocks were sectioned to 4-micron thick slices and mounted to slides. After deparaffinization and rehydration, 3% H_2_O_2_ was used to block endogenous peroxidase for 15 minutes. Then, antigen retrieval was accomplished by heating slices for 10 minutes within Tris-EDTA buffer (pH = 9.0) and slides were blocked in 2.5% goat serum for one hour.

The expression of mucins was detected by using the following primary monoclonal antibodies (MAbs) which were purchased from Abcam company: ab109185 (recombinant MAb to MUC1), ab134119 (recombinant Mab to MUC2), and ab3649 (recombinant MAb to MUC5AC). Primary antibodies were diluted against 2.5% goat serum according to instructions and were incubated with tumour tissue slides overnight at 4°C. The next day, the sections were incubated with secondary antibodies (GTVisionTM III Detection System/Mo&Rb, GK500710, Gene Tech Company) for one hour; then, 3,3-diaminobenzidine was used to coloration with counterstaining of hematoxylin. Sections were dehydrated in ethanol and xylene and reembedded in neutral resin before observation under microscopy.

Tumours were classified into three histology grades according to their heterogeneity, differentiation level, and nuclear split phases: low grade, moderate grade, and high grade. The expression of mucin was classified as negative or positive, and positive expression was only considered when reaction products localized in the expected cellular component of tumour cells. One positive tumour cell was sufficient for diagnosing. Slides were excluded from the following analyses if they had unsatisfactory tissue quality such as tissue tears or folds.

The results of IHC were assessed by two experienced pathologists. When the two pathologists got different results, the third pathologist participated in the discussion and came to the final conclusion or abandoned uncertain results. The pathologists evaluating the MUC staining were blinded to patients' outcomes to minimize bias.

### 2.4. Statistical Analysis

Pearson's *χ*^2^ test and Fisher's exact test were used to analyze the correlations between mucin expression and major baseline and clinicopathological characteristics. Continuous variables (mucin's expression level) were dichotomized by optimal cut-off values calculated by the survminer R package (version 0.4.9). The Kaplan-Meier method was used to plot survival curves; log-rank (Mantel-Cox) tests were used to compare the difference between groups; and the Mantel–Haenszel method was used to calculate the HR. Cox proportional hazard models and logistic regression models were used to make multivariate analyses, and parameters with a *p* value less than 0.10 in the univariate analyses were included in the multivariate analyses. All statistics were analyzed by SPSS 26.0 software (SPSS, Inc., Chicago, IL). All *p* values are two-sided, and differences with *p* values less than 0.05 were considered statistically significant.

This study was approved by the Ethics Board of Shanghai Cancer Center, Fudan University, and all the involved patients provided informed consent for their personal data being used for research purposes.

## 3. Results

### 3.1. Typical Mucin Expression Pattern in PDAC

**Figure 1:**
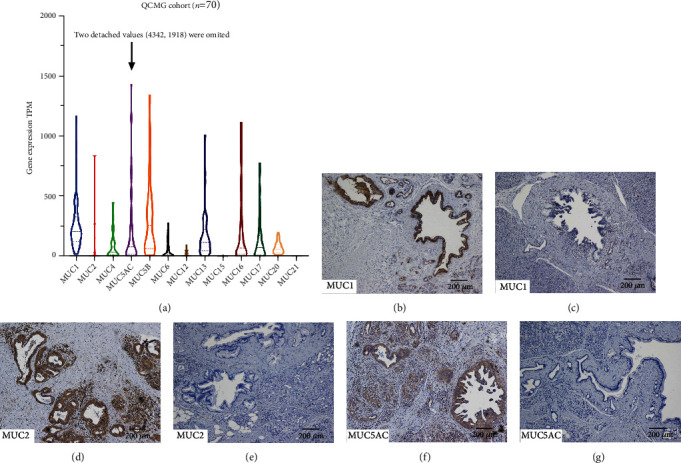
The expression patterns of mucins in PDAC tissues. (a) mRNA expression level of mucins in the QCMG PDAC cohort (*n* = 70). (b–g) Low-power-field images showing the representative positive and negative results of IHC staining mucins' expression in the FUSCC PDAC cohort. Corresponding high-power-field images were displayed in Supplementary Figure 1. (b, c) MUC1-positive expression was only considered when positive coloration located in the apical membrane of PDAC cells; (d, e) MUC2-positive expression was only considered when positive coloration was located in the cytoplasm of PDAC cells. (f, g) MUC5AC-positive expression was only considered when positive coloration was located in the cytoplasm of PDAC cells. Abbreviations: TPM: transcripts per million reads; PDAC: pancreatic ductal adenocarcinoma.

The mucin expression pattern is altered throughout the progression and metastasis of PDAC [9, 18], and MUC1 positive, MUC2 negative, and MUC5AC positive were regarded as the typical mucin expression pattern in PDAC [19, 20]. However, this typical expression pattern was obtained by analyzing IHC results, which may be confounded by antibodies' efficiency and pathologists' professionalism. To verify this typical mucin expression pattern, we analyzed the mucin expression in the QCMG PDAC cohort (Figure 1(a)). The median TPM expression level of MUC1, MUC2, and MUC5AC were 251.4, 1.470, and 80.93, respectively. In addition, MUC5B, MUC13, MUC16, MUC17, and MUC20 also had comparatively high expression in PDAC, and MUC4, MUC6, MUC12, MUC15, and MUC21 were commonly absent in PDAC.

### 3.2. Baseline and Clinicopathological Characteristics

The median age of investigated patients of the FUSCC cohort was 61.9 years old (30 to 84 years old). Females comprised 42.9% of the cohort, and males comprised the rest. We selected MUC1, MUC2, and MUC5AC for further investigation because they were routinely stained in the postoperative pathological reports of FUSCC for antidiastole and grading of intraductal papillary mucinous neoplasms [21]. Among 427 investigated PDAC specimens, 92.0% (392/426) were MUC1 positive, 16.3% (69/424) were MUC2 positive, and 88.6% (365/412) were MUC5AC positive, which was consistent with the typical mucin expression pattern. Representative images of immunohistology coloration are shown in Figure 1 and Supplementary Figure 1. Baseline and clinicopathological characteristics are summarized in Tables 1–3.

When patients were divided into subgroups based on MUC1 expression, MUC2 expression, or MUC5AC expression, there were no significant differences in terms of age, sex, tumour grade, tumour size, N stage, tumour location, vascular invasion, and diabetes mellitus history. Results with statistical significance were as follows: MUC1 absence was correlated with a higher proportion of TNM stage III PDAC (26.5% versus 11.0%, *p* = 0.011), and MUC1 absence and MUC2 presence were associated with perineural infiltration (*p* = 0.011 and *p* = 0.03, respectively).

### 3.3. MUC1 Absence and MUC2 Presence Indicate a Shorter Overall Survival

**Figure 2:**
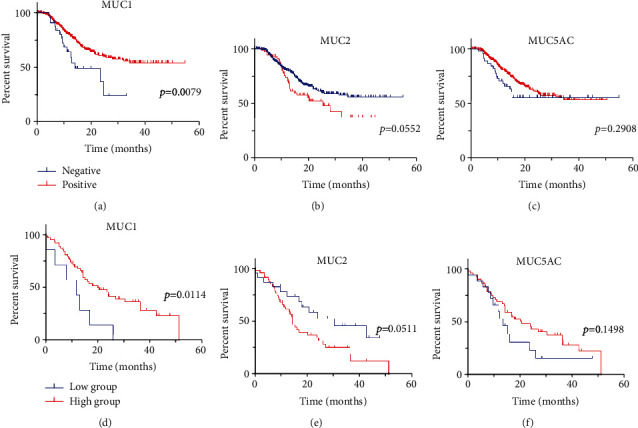
Survival curves of PDAC patients in relevant to mucin expression. The survival plots were plotted by Kaplan-Meier's method, and Mantel-Cox tests were used to compare the curves. (a–c) FUSCC cohort was stratified by mucins' IHC staining results. (a) MUC1-negative patients (*n* = 34) had worse survival compared to MUC1-positive patients (*n* = 392). (b) MUC2-positive patients (*n* = 69) tended to survive a shorter time compared to MUC2-negative patients (*n* = 355). (c) There was no statistical significance of overall survival between the subgroups stratified by MUC5AC expression (*n* = 365 and *n* = 47, respectively). (d) The cut-off value of 77.90 TPM stratified the QCMG cohort into the high MUC1 expression subgroup (*n* = 63) and the low MUC1 expression subgroup (*n* = 7) with a significant difference in OS. (e) The cut-off value of 0.682 TPM stratified the QCMG cohort into the high MUC2 expression subgroup (*n* = 47) and the low MUC2 expression subgroup (*n* = 23) with a marginally significant difference in OS. (f) The cut-off value of 17.83 TPM stratified the QCMG cohort into the high MUC5AC expression subgroup (*n* = 52) and the low MUC5AC expression subgroup (*n* = 18); although the survival curves had a separating tendency in the early stage, there was no statistical OS difference. Abbreviations: PDAC: pancreatic ductal adenocarcinoma.

**Figure 3:**
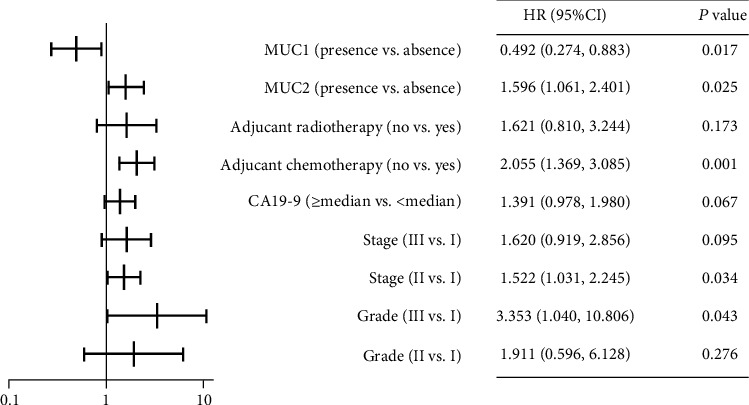
Multivariate analyses of mucin expression and clinicopathological characteristics on overall survival. The univariate analysis results of the FUSCC cohort are displayed in Supplementary Table 2, and risk factors with a *p* value less than 0.1 in the univariate analysis were integrated to make multivariate logistic regression. The *X*-axis of this forest plot stands for HR, which was calculated by logistic Cox regression, enter method. The median of CA19-9 was 188.6 U/ml. Abbreviations: HR: hazard ratio; CI: confidence interval.

**Figure 4:**
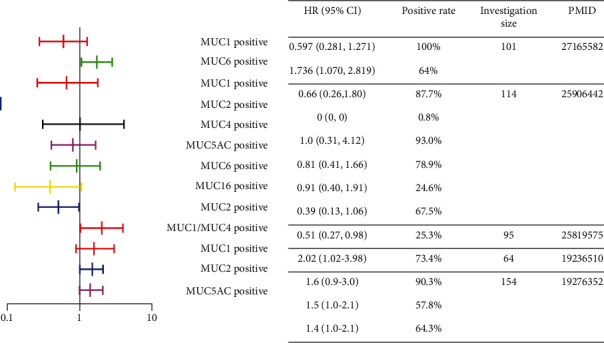
Previous studies on mucins' prognostic values in PDAC. Five investigations with reported HR values were included in this forest plot. The *X*-axis stands for HR. Abbreviations: HR: hazard ratio; CI: confidence interval.

At the end of the follow-up period, 31.4% (134/427) of the FUSCC PDAC cohort had died. The median follow-up span was 414 days (21 to 1641 days). Patients without MUC1 expression had a shorter OS (Figure 2(a), HR = 0.374, *p* = 0.0079), and patients with MUC2 expression had a shorter OS with marginal significance (Figure 3(b), HR = 1.558, *p* = 0.0552). The mean survival time of MUC1-positive and MUC1-negative patients was 1094 days (95% confidence interval CI: 1016 to 1173 days) and 571 days (95% CI: 440 to 703 days), respectively. The mean survival time of MUC2-positive and MUC2-negative patients was 792 days (95% CI: 659 to 924 days) and 1108 days (95% CI: 1024 to 1193 days), respectively. MUC5AC absence tended to correlate with short-term death but was not significantly associated with long-term survival (Figure 4(c), HR = 0.7381, *p* = 0.2714).

Regarding the QCMG PDAC cohort, the survminer R package output of the optimal cut-off values for MUC1, MUC2, and MUC5AC was 77.9 TPM, 0.68 TPM, and 17.8 TPM, respectively. The mean survival time of the MUC1 high-expressed and low-expressed group was 763 days (95% CI: 619 to 907 days) and 341 days (95% CI: 150 to 531 days), respectively, and the mean survival time of the MUC2 high-expressed and low-expressed group was 613 days (95% CI: 465 to 760 days) and 869 days (95% CI: 642 to 1096 days), respectively. MUC1 high-expression or MUC2 low-expression was associated with longer OS (Figures 2(d) and 2(e), HR = 0.2117 and 1.817, *p* = 0.0114 and 0.0511, respectively). Patients' subgroups stratified by MUC5AC expression did not have an OS difference, but short-term death tended to occur more in the MUC5AC low-expressed subgroup (Figure 2(f), HR = 0.5838, *p* = 0.1498).

We then stratified the QCMG PDAC cohort by other mucins' expression and performed survival analysis (Supplementary Table 1). MUC4, MUC12, MUC16, MUC16, and MUC20 were also significantly associated with PDAC patients' survival and warranted further investigation.

### 3.4. MUC1 Absence and MUC2 Presence Are Independent Risk Factors for Overall Survival in PDAC Patients

The results of univariate analysis of the FUSCC cohort are summarized in Supplementary Table 2. Risk factors with a *p* value less than 0.1, i.e., tumour grade, tumour stage, CA19-9 level, adjuvant chemotherapy, adjuvant radiotherapy, MUC1 expression, and MUC2 expression, were integrated to make multivariate analysis (Figure 3). The multivariable-adjusted Cox regression model showed that the HR for mortality comparing patients with those without MUC1 expression was 0.492 (95% CI: 0.274 to 0.883, *p* = 0.017), and mortality comparing patients with those without MUC2 expression was 1.596 (95% CI: 1.061 to 2.401, *p* = 0.025). Therefore, MUC1 absence and MUC2 presence were considered as independent risk factors for prognosticating survival time of PDAC patients after surgical section. High tumour grade, high tumour stage, and not receiving adjuvant chemotherapy were also independently correlated with increased mortality.

## 4. Discussion

PDAC is a malignancy characterized by high mortality and unsatisfactory survival. Its 5-year survival rate is very low, and the recurrence and metastasis rates are high. Therefore, it is necessary to find reliable prognostic markers, which can not only predict the survival rate of patients but also help to find potential therapeutic targets.

One of the remarkable features of PDAC is the abundant dense stroma [22], which enriches multiple aberrantly expressed mucins and merit investigation. Emerging roles of mucins are discovered in the progression, development, and metastasis of malignancies, including intestinal cancer, ovarian cancer, and haematological malignancies [23]. Overexpressed membrane-bound mucins interact with receptor tyrosine kinases such as epidermal growth factor receptor (EGFR) and attenuate signalling pathways downstream of transforming growth factor-*α* (TGF-*α*) and EGFR [24] and hence play protective roles for cancer cells. Therefore, membrane-bound MUC1 is generally recognized as an oncoprotein in epithelial cancers [11, 25]. However, the function of MUC1 can be switched depending on its glycosylation status, based on which MUC1 has a dual function of proinflammatory and anti-inflammatory factors [26]. In addition, MUC1 absence is associated with altered tumour microenvironment (TME). MUC1-deficient PDAC exhibits significant different immune reaction compared to wildtype PDAC in mouse models [27], and MUC1 absence results in the proliferation and activation of myeloid-derived suppressor cells (MDSC) and regulatory T cells (Treg), which correspond to the immunosuppressive tumour microenvironment and are responsible for tumour immune evasion [28]. Besides, transmembrane mucins contribute to the junction and the polarity of epithelial cell, loss of which promotes malignant epithelial-mesenchymal transition (EMT) and tumorigenesis, and downregulation of membrane-bound mucins doubtlessly reduces the immunogenicity of tumour cells.

Secreted mucins, such as MUC2, form a protective mucus barrier and help epithelial cells get rid of inflammation and tumorigenesis in physiological condition. Paradoxically, MUC2 has increased expression level in certain types of gastrointestinal malignancies [23, 29], which denotes that MUC2 may also be employed by cancer cells and function as a mucous barrier against antitumour immune reaction. MUC5AC is another secreted mucin and promotes KLF4-mediated PDAC cancerous stemness [9]. In addition, CA19-9, the most commonly used prognostic marker for pancreatic cancerous disease, is present on the surface of MUC1 and MUC5AC [22, 30]; with respect to the recent discovery that CA19-9 supports the initiation and progression of PDAC [31], the interaction between mucins and CA19-9 suggests that mucins are not only prognostic factors but also participate in the onset and advancement of PDAC.

There was a PDAC subgroup featured by MUC1-negative, MUC2-positive, or MUC5AC-negative expression patterns, and this atypical subgroup accounts for 31.2% of the total FUSCC PDAC cohort; hence, it is necessary to study these PDAC patients with atypical mucin expression to clarify the association between mucin expression and prognosis of PDAC patients.

However, other researchers had contradictory conclusions about the correlation between mucin expression and PDAC patients' prognosis [32]. Previous investigations about the prognostic values of mucins with calculated HRs are summarized in Figure 4 [20, 29, 33, 34, 35]. Besides, Hinoda et al. surveyed 70 advanced PDAC patients and found that 55.7% of patients with MUC1 presence had shorter survival [36]. Pantano et al. researched 59 radically resected PDAC patients and drew the conclusion that the MUC2-positive subgroup (10.2%) had longer survival, and MUC5AC did not have a prognostic value [37].

The unstable conclusions drawn from the above studies can be attributed to insufficient investigated PDAC patients and unexpected mucin-positive rate, so their cohorts are not representative. Therefore, it is vital to use a larger cohort to elucidate the correlation between mucin expression and clinical outcomes of PDAC patients. In this study, we used a relatively large cohort and discovered that MUC1 absence and MUC2 presence were associated with worse OS in PDAC patients. After controlling for age, gender, tumour location, tumour grade, tumour stage, perineural invasion, vascular thrombi, diabetes mellitus history, baseline CA19-9 serum level, adjuvant chemotherapy, and adjuvant radiotherapy treatment history, MUC1 absence and MUC2 presence were identified as independent risk factors. Although MUC5AC did not show a prognostic value, we noticed that the MUC5AC-negative group had more death events compared to the MUC5AC-positive group in the early stage of following-up.

Our results resolve the above controversy. We conclude that MUC1 absence is an independent risk factor for PDAC in the Chinese population. Further studies are needed to elucidate the effect of MUC1 glycosylation on PDAC. Our study also suggested that the existence of MUC2 predicts a worse survival rate in PDAC, which may be the clinical evidence that cancer cells exploit MUC2 to form a protective mucous barrier to evade from immune attack.

Our research focuses on the resected and pathologically diagnosed PDAC, which makes our study more homogenous. In addition, our research has a relatively large cohort consisting of 427 PDAC patients, which makes our results more representative and convincing. The retrospective design becomes the major limitation of our study. Besides, since our research is a surgical cohort, patients with unresectable PDAC were excluded. We hope our clinical findings contribute to future exploration of PDAC-targeted therapy.

## 5. Conclusion

Atypical mucin expression patterns, i.e., MUC1 absence or MUC2 presence, prognosticate shorter OS time in PDAC patients. MUC5AC absence did not predict PDAC patients' OS but was correlated with short-term death.

## Figures and Tables

**Table 1:** Clinicopathological characteristics of FUSCC PDAC patients stratified by MUC1 expression.

Variables	MUC1 negative	MUC1 positive	*p*
Age, (*n*%)			0.303
<62	19 (55.9%)	183 (46.7%)	
≥62	15 (44.1%)	209 (53.3%)	
Sex, (*n*%)			0.110
Male	24 (70.6%)	218 (56.5%)	
Female	10 (29.4%)	168 (43.5%)	
Tumour grade, (*n*%)			0.534
Low	2 (5.9%)	17 (4.4%)	
Moderate	23 (67.6%)	230 (59.7%)	
High	9 (26.5%)	138 (35.8%)	
Tumour stage, (*n*%)			0.011
I	15 (44.1%)	154 (39.3%)	
II	10 (29.4%)	195 (49.7%)	
III	9 (26.5%)	43 (11.0%)	
T stage, (*n*%)			0.591
T1	8 (23.5%)	67 (17.1%)	
T2	19 (55.9%)	225 (57.4%)	
T3	7 (20.6%)	100 (25.5%)	
N stage, (*n*%)			0.499
N0	19 (55.9%)	197 (51.0%)	
N1	10 (29.4%)	149 (38.6%)	
N2	5 (14.7%)	40 (10.4%)	
Tumour location, (*n*%)			0.222
Head	20 (58.8%)	210 (54.4%)	
Body	13 (38.2%)	174 (45.1%)	
Tail	1 (2.9%)	2 (0.5%)	
Vascular invasion, (*n*%)			0.681
No	10 (29.4%)	101 (26.2%)	
Yes	24 (70.6%)	285 (73.8%)	
Perineural infiltration, (*n*%)			0.011
No	25 (73.5%)	342 (88.6%)	
Yes	9 (26.5%)	44 (11.4%)	
Diabetes mellitus, (*n*%)			0.226
No	26 (76.5%)	326 (84.5%)	
Yes	8 (23.5%)	60 (15.5%)	

**Table 2:** Clinicopathological characteristics of FUSCC PDAC patients stratified by MUC2 expression.

Variables	MUC2 negative	MUC2 positive	*p*
Age, (*n*%)			0.905
<62	167 (47.0%)	33 (47.8%)	
≥62	188 (53.0%)	36 (52.2%)	
Sex, (*n*%)			0.760
Male	201 (57.4%)	41 (59.4%)	
Female	149 (42.6%)	28 (40.6%)	
Tumour grade, (*n*%)			0.171
Low	13 (3.7%)	6 (8.7%)	
Moderate	214 (61.3%)	38 (55.1%)	
High	122 (35.0%)	25 (36.2%)	
Tumour stage, (*n*%)			0.699
I	143 (40.3%)	26 (37.7%)	
II	167 (47.0%)	36 (52.2%)	
III	45 (12.7%)	7 (10.1%)	
T stage, (*n*%)			0.730
T1	64 (18.0%)	10 (14.5%)	
T2	204 (57.5%)	40 (58.0%)	
T3	87 (24.5%)	19 (27.5%)	
N stage, (*n*%)			0.746
N0	182 (52.0%)	33 (47.8%)	
N1	130 (37.1%)	29 (42.0%)	
N2	38 (10.9%)	7 (10.1%)	
Tumour location, (*n*%)			0.407
Head	191 (54.6%)	39 (56.5%)	
Body	158 (45.1%)	29 (42.0%)	
Tail	1 (0.3%)	1 (1.4%)	
Vascular invasion, (*n*%)			0.830
No	92 (26.3%)	19 (27.5%)	
Yes	258 (73.7%)	50 (72.5%)	
Perineural infiltration, (*n*%)			0.030
No	312 (89.1%)	55 (79.7%)	
Yes	38 (10.9%)	14 (20.3%)	
Diabetes mellitus, (*n*%)			0.174
No	297 (84.9%)	54 (78.3%)	
Yes	53 (15.1%)	15 (21.7%)	

**Table 3:** Clinicopathological characteristics of FUSCC PDAC patients stratified by MUC5AC expression.

Variables	MUC5AC negative	MUC5AC positive	*p*
Age, (*n*%)			0.486
<62	20 (42.6%)	175 (47.9%)	
≥62	27 (57.4%)	190 (52.1%)	
Sex, (*n*%)			0.145
Male	32 (68.1%)	205 (56.9%)	
Female	15 (31.9%)	155 (43.1%)	
Tumour grade, (*n*%)			0.120
Low	0 (0.0%)	19 (5.3%)	
Moderate	26 (55.3%)	220 (61.3%)	
High	21 (44.7%)	120 (33.4%)	
Tumour stage, (*n*%)			0.067
I	19 (40.4%)	146 (40.0%)	
II	27 (57.4%)	170 (46.6%)	
III	1 (2.1%)	49 (13.4%)	
T stage, (*n*%)			0.958
T1	8 (17.0%)	63 (17.3%)	
T2	28 (59.6%)	210 (57.5%)	
T3	11 (23.4%)	92 (25.2%)	
N stage, (*n*%)			0.084
N0	21 (44.7%)	189 (52.5%)	
N1	24 (51.1%)	130 (36.1%)	
N2	2 (4.3%)	41 (11.4%)	
Tumour location, (*n*%)			0.058
Head	20 (42.6%)	203 (55.6%)	
Body	26 (55.3%)	156 (43.3%)	
Tail	1 (2.1%)	1 (0.3%)	
Vascular invasion, (*n*%)			0.345
No	10 (21.3%)	100 (27.8%)	
Yes	37 (78.7%)	260 (72.2%)	
Perineural infiltration, (*n*%)			0.354
No	39 (83.0%)	316 (87.8%)	
Yes	8 (17.0%)	44 (12.2%)	
Diabetes mellitus, (*n*%)			0.097
No	36 (76.6%)	309 (85.8%)	
Yes	11 (23.4%)	51 (14.2%)	

## Data Availability

The datasets used and analyzed during this study are available from the corresponding author upon reasonable request.

## Abbreviations

AJCC: The American Joint Committee on Cancer
CA153: Carbohydrate antigen 153
CA19-9: Carbohydrate antigen 19-9
CI: Confidence interval
EGFR: Epidermal growth factor receptor
EMT: Epithelial-mesenchymal transition
HR: Hazard ratio
IHC: Immunohistochemistry
IPMN: Intraductal papillary mucinous neoplasm
Mab: Monoclonal antibody
MCN: Mucinous cystic neoplasm
MDSC: Myeloid-derived suppressor cells
OS: Overall survival
PDAC: Pancreatic ductal adenocarcinoma
TGF-*α*: Transforming growth factor-*α*
TME: Tumour microenvironment
Treg: Regulatory T cells.

## Supplementary Materials

Supplementary Materials Supplementary Figure 1 displayed high-power-field images of IHC staining results in the FUSCC cohort. Supplementary Table 1 displayed mucins' expression in correlation to PDAC patients' OS in the QCMG cohort. Supplementary Table 2 displayed univariate and multivariate analyses of OS in the FUSCC cohort.
