# Supplementary material for: Atypical Mucin Expression Predicts Worse Overall Survival in Resectable Pancreatic Ductal Adenocarcinoma
Source: J Immunol Res. 2022 Jul 21;2022:7353572. doi: 10.1155/2022/7353572 (PMC9334048; doi:10.1155/2022/7353572)
Supplement: Supplementary Materials — Supplementary Figure 1 displayed high-power-field images of IHC staining results in the FUSCC cohort. Supplementary Table 1 displayed mucins' expression in correlation to PDAC patients' OS in the QCMG cohort. Supplementary Table 2 displayed univariate and multivariate analyses of OS in the FUSCC cohort. [file 7353572.f1.zip › Supplementary Figure 1.doc]

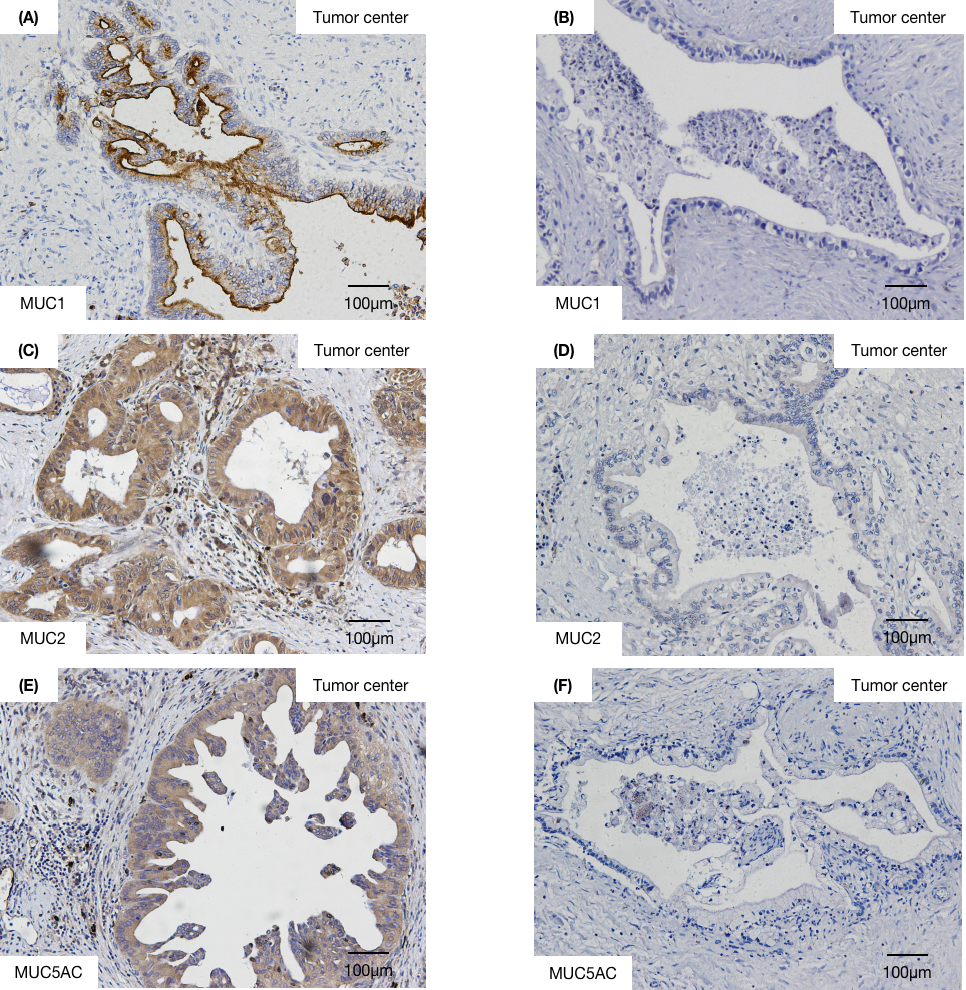


**Supplementary Figure 1. Staining mucins’ expression in PDAC tissues.**

High-power-field images showing the representative IHC staining results in FUSCC PDAC specimens. A, B: MUC1 positive and negative staining results. C, D: MUC2 positive and negative staining results. E, F: MUC5AC positive and negative staining results.
