# Supplementary material for: Atypical Mucin Expression Predicts Worse Overall Survival in Resectable Pancreatic Ductal Adenocarcinoma
Source: J Immunol Res. 2022 Jul 21;2022:7353572. doi: 10.1155/2022/7353572 (PMC9334048; doi:10.1155/2022/7353572)
Supplement: Supplementary Materials — Supplementary Figure 1 displayed high-power-field images of IHC staining results in the FUSCC cohort. Supplementary Table 1 displayed mucins' expression in correlation to PDAC patients' OS in the QCMG cohort. Supplementary Table 2 displayed univariate and multivariate analyses of OS in the FUSCC cohort. [file 7353572.f1.zip › Supplementary Materials.doc]

**Supplementary Materials**


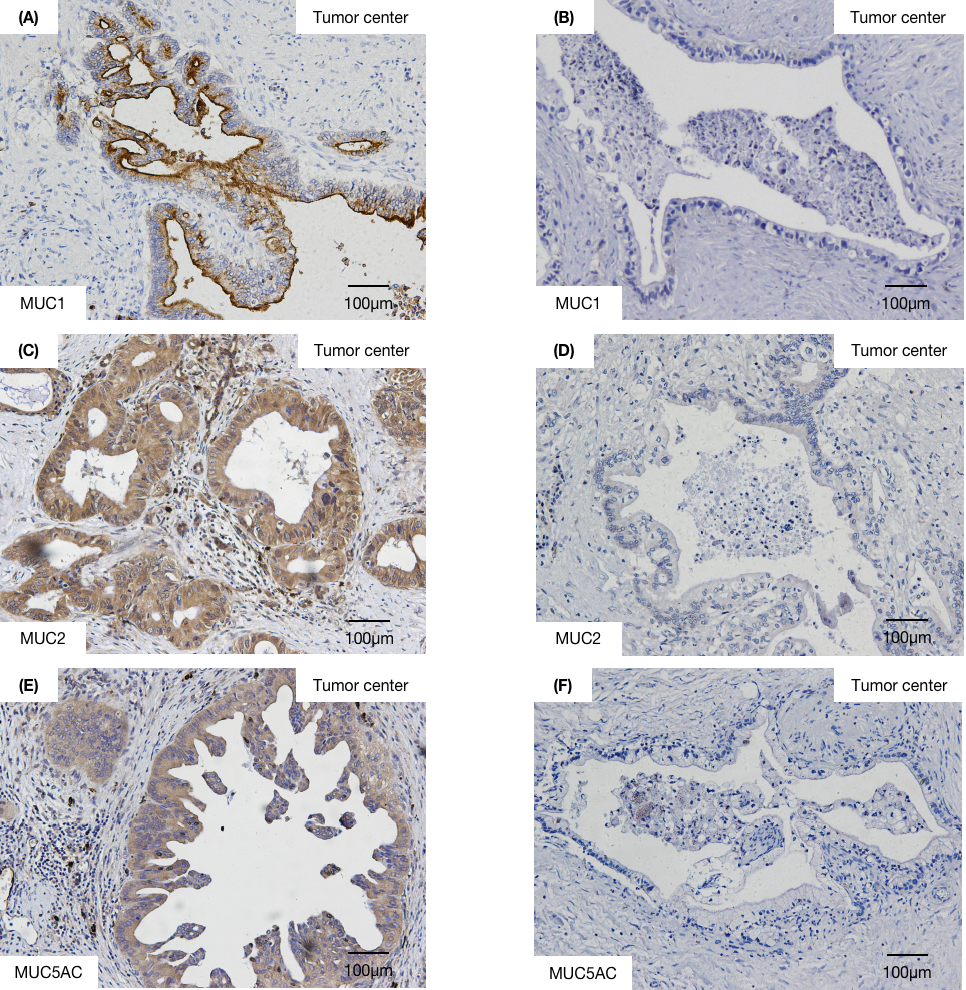


**Supplementary Figure 1. Staining mucins’ expression in PDAC tissues.**

High-power-field images showing the representative IHC staining results in FUSCC PDAC specimens. A, B: MUC1 positive and negative staining results. C, D: MUC2 positive and negative staining results. E, F: MUC5AC positive and negative staining results.

| Characteristics | Parameters | Cut-off value (TPM) | HR | 95% CI | p value |
| --- | --- | --- | --- | --- | --- |
| MUC1 | high -expressed | 77.90 | 1 |  |  |
|  | low-expressed |  | 0.212 | 0.064 to 0.705 | **0.0114** |
| MUC2 | high -expressed | 0.68 | 1 |  |  |
|  | low-expressed |  | 1.817 | 0.997 to 3.309 | 0.0511 |
| MUC4 | high -expressed | 2.68 | 1 |  |  |
|  | low-expressed |  | 2.397 | 1.073 to 5.355 | **0.0330** |
| MUC5AC | high -expressed | 17.83 | 1 |  |  |
|  | low-expressed |  | 0.584 | 0.281 to 1.214 | 0.1498 |
| MUC5B | high -expressed | 19.56 | 1 |  |  |
|  | low-expressed |  | 2.194 | 0.922 to 5.222 | 0.0757 |
| MUC6 | high -expressed | 45.62 | 1 |  |  |
|  | low-expressed |  | 0.616 | 0.330 to 1.150 | 0.1280 |
| MUC12 | high -expressed | 2.37 | 1 |  |  |
|  | low-expressed |  | 2.193 | 1.131 to 4.253 | **0.0202** |
| MUC13 | high -expressed | 34.03 | 1 |  |  |
|  | low-expressed |  | 0.240 | 0.093 to 0.621 | **0.0033** |
| MUC15 | high -expressed | 0.17 | 1 |  |  |
|  | low-expressed |  | 1.489 | 0.832 to 2.666 | 0.1805 |
| MUC16 | high -expressed | 42.92 | 1 |  |  |
|  | low-expressed |  | 2.795 | 1.543 to 5.062 | **0.0007** |
| MUC17 | high -expressed | 1.40 | 1 |  |  |
|  | low-expressed |  | 0.348 | 0.117 to 1.033 | 0.0573 |
| MUC20 | high -expressed | 19.13 | 1 |  |  |
|  | low-expressed |  | 0.281 | 0.082 to 0.958 | **0.0425** |
| MUC21 | high -expressed | 0.47 | 1 |  |  |
|  | low-expressed |  | 1.939 | 0.804 to 4.676 | 0.1402 |

**Supplementary Table 1. Mucins’ expression in correlation to PDAC patients’ OS in QCMG cohort.**

| Characteristics | Parameters | Univariate Analysis | | | Multivariate Analysis (Enter Method) | | |
| --- | --- | --- | --- | --- | --- | --- | --- |
|  |  | HR | 95% CI | p value | HR | 95% CI | p value |
| Age | < Median (63 years) | 1 |  |  |  |  |  |
|  | ≥ Median (63 years) | 1.051 | 0.747 to 1.478 | 0.776 |  |  |  |
| Sex | Male | 1 |  |  |  |  |  |
|  | Female | 1.061 | 0.752 to 1.496 | 0.737 |  |  |  |
| Tumour Location | Head and Neck of Pancreas | 1 |  |  |  |  |  |
|  | Body of Pancreas | 0.970 | 0.686 to 1.372 | 0.865 |  |  |  |
|  | Tail of Pancreas | 2.212 | 0.542 to 9.028 | 0.268 |  |  |  |
| Tumour Grade | Grade I | 1 |  |  | 1 |  |  |
|  | Grade II | 2.002 | 0.630 to 6.366 | 0.240 | 1.911 | 0.596 to 6.128 | 0.276 |
|  | Grade III | 3.631 | 1.136 to 11.605 | **0.030** | 3.353 | 1.040 to 10.806 | **0.043** |
| Tumour Stage | Stage I | 1 |  |  | 1 |  |  |
|  | Stage II | 1.725 | 1.176 to 2.532 | **0.005** | 1.522 | 1.031 to 2.245 | **0.034** |
|  | Stage III | 1.918 | 1.113 to 3.307 | **0.019** | 1.620 | 0.919 to 2.856 | 0.095 |
| Perineural Invasion | No | 1 |  |  |  |  |  |
|  | Yes | 0.825 | 0.495 to 1.372 | 0.458 |  |  |  |
| Vascular Tumour Thrombi | No | 1 |  |  |  |  |  |
|  | Yes | 0.811 | 0.556 to 1.182 | 0.276 |  |  |  |
| Diabetes Mellitus | No | 1 |  |  |  |  |  |
|  | Yes | 0.993 | 0.610 to 1.614 | 0.976 |  |  |  |
| CA19-9 Level | < Median (188.6 U/ml) | 1 |  |  | 1 |  |  |
|  | ≥ Median (188.6 U/ml) | 1.519 | 1.077 to 2.143 | **0.017** | 1.391 | 0.978 to 1.980 | 0.067 |
| Adjuvant Chemotherapy | Yes | 1 |  |  | 1 |  |  |
|  | No | 2.032 | 1.367 to 3.022 | **< 0.001** | 2.055 | 1.369 to 3.085 | **0.001** |
| Adjuvant Radiotherapy | Yes | 1 |  |  | 1 |  |  |
|  | No | 1.937 | 0.983 to 3.816 | **0.056** | 1.621 | 0.810 to 3.244 | 0.173 |
| MUC1 | negative | 1 |  |  |  |  |  |
|  | positive | 0.488 | 0.284 to 0.839 | **0.009** | 0.492 | 0.274 to 0.883 | **0.017** |
| MUC2 | negative | 1 |  |  |  |  |  |
|  | positive | 1.479 | 0.988 to 2.214 | **0.057** | 1.596 | 1.061 to 2.401 | **0.025** |
| MUC5AC | negative | 1 |  |  |  |  |  |
|  | positive | 0.761 | 0.466 to 1.241 | 0.273 |  |  |  |

**Supplementary Table 2. Univariate and multivariate analyses of overall survival with mucins’ expression and clinicopathological characteristics.**
