# Supplementary material for: Atypical Mucin Expression Predicts Worse Overall Survival in Resectable Pancreatic Ductal Adenocarcinoma
Source: J Immunol Res. 2022 Jul 21;2022:7353572. doi: 10.1155/2022/7353572 (PMC9334048; doi:10.1155/2022/7353572)
Supplement: Supplementary Materials — Supplementary Figure 1 displayed high-power-field images of IHC staining results in the FUSCC cohort. Supplementary Table 1 displayed mucins' expression in correlation to PDAC patients' OS in the QCMG cohort. Supplementary Table 2 displayed univariate and multivariate analyses of OS in the FUSCC cohort. [file 7353572.f1.zip › Supplementary Table 1.doc]

| Characteristics | Parameters | Cut-off value (TPM) | HR | 95% CI | p value |
| --- | --- | --- | --- | --- | --- |
| MUC1 | high -expressed | 77.90 | 1 |  |  |
|  | low-expressed |  | 0.212 | 0.064 to 0.705 | **0.0114** |
| MUC2 | high -expressed | 0.68 | 1 |  |  |
|  | low-expressed |  | 1.817 | 0.997 to 3.309 | 0.0511 |
| MUC4 | high -expressed | 2.68 | 1 |  |  |
|  | low-expressed |  | 2.397 | 1.073 to 5.355 | **0.0330** |
| MUC5AC | high -expressed | 17.83 | 1 |  |  |
|  | low-expressed |  | 0.584 | 0.281 to 1.214 | 0.1498 |
| MUC5B | high -expressed | 19.56 | 1 |  |  |
|  | low-expressed |  | 2.194 | 0.922 to 5.222 | 0.0757 |
| MUC6 | high -expressed | 45.62 | 1 |  |  |
|  | low-expressed |  | 0.616 | 0.330 to 1.150 | 0.1280 |
| MUC12 | high -expressed | 2.37 | 1 |  |  |
|  | low-expressed |  | 2.193 | 1.131 to 4.253 | **0.0202** |
| MUC13 | high -expressed | 34.03 | 1 |  |  |
|  | low-expressed |  | 0.240 | 0.093 to 0.621 | **0.0033** |
| MUC15 | high -expressed | 0.17 | 1 |  |  |
|  | low-expressed |  | 1.489 | 0.832 to 2.666 | 0.1805 |
| MUC16 | high -expressed | 42.92 | 1 |  |  |
|  | low-expressed |  | 2.795 | 1.543 to 5.062 | **0.0007** |
| MUC17 | high -expressed | 1.40 | 1 |  |  |
|  | low-expressed |  | 0.348 | 0.117 to 1.033 | 0.0573 |
| MUC20 | high -expressed | 19.13 | 1 |  |  |
|  | low-expressed |  | 0.281 | 0.082 to 0.958 | **0.0425** |
| MUC21 | high -expressed | 0.47 | 1 |  |  |
|  | low-expressed |  | 1.939 | 0.804 to 4.676 | 0.1402 |

**Supplementary Table 1. Mucins’ expression in correlation to PDAC patients’ OS in QCMG cohort.**
